# Why Are Foodies Active on Social Network Services? An Exploratory Study on Foodies’ Influence on Social Media

**DOI:** 10.3390/foods13213476

**Published:** 2024-10-30

**Authors:** Jin A Jang, EunJung Lee, Hyosun Jung

**Affiliations:** 1 Department of Wellness Food Therapy, Ansan University, Ansan-si 15328, Republic of Korea; 2 Food and Nutrition Major, School of Wellness Industry Convergence, Hankyong National University, Jungang-no, Anseong-si 17579, Republic of Korea; 3 Center for Converging Humanities KyungHee University, Seoul 02447, Republic of Korea; chefcook@khu.ac.kr

**Keywords:** gastronome, foodie, social media, social network service, dining out

## Abstract

As social media continues to gain traction in the food industry, this study explored how cooking and dining-out behaviors and social media use differ depending on the foodie tendencies of consumers, particularly Korean Millennial and Gen Z consumers. Additionally, this study considered the effect of foodie tendencies on active social media usage. Based on the Foodie Index, two groups were identified: high and low. The high group scored higher than the low group on foodie knowledge and spending, food interest, and time commitment. Comparison of the food, cooking, and dining-out behaviors of both groups revealed a significantly greater frequency of cooking and a higher proportion of using their own ingredients, even when dining alone, in the high group. Analysis of social media use characteristics also demonstrated significantly higher scores for the high group than the low group in terms of the degree of use, average daily usage time, checking frequency, and use of social media recommendations when cooking and purchasing food. Furthermore, foodie inclination significantly influenced active usage behavior, as users shared informative content and frequently posted articles and photos. This study found that foodies play a leading role in producing food-related information by actively using and sharing social media. Considering this ripple effect, consumers’ foodie tendencies can be used as an important measure in food marketing-related research.

## 1. Introduction

Social media is a form of electronic communication that allows users to create virtual communities to share information, ideas, messages, and other content such as pictures and videos. Popular social media platforms include Facebook, Twitter, YouTube, Instagram, Snapchat, Pinterest, and TikTok. Social media is commonly used to maintain and develop relationships with friends, communicate and interact with others, and seek information and entertainment [1,2,3]. The rise of social media has shaped how consumers interact with food [4]. Green et al. [5] define foodies as individuals who try new restaurants, new recipes, and cooking with local ingredients; attend food and drink festivals; try food from other cultures; share dining-out or cooking experiences online through blogs; and travel for new food-related experiences. In other words, foodies are passionate about the pursuit of good food and take pleasure in dining out and exploring food [6,7]. About 66% of foodies share their food preferences on social networks, and more than a third say they cook to impress others [6]. In particular, foodies use highly visual social media platforms such as Facebook, Pinterest, and Instagram to demonstrate their food and cooking skills [6]. Food blogs are also a popular platform upon which chefs share their recipes, knowledge, experiences, and opinions and engage in online communication with other consumers. In their survey, Foodista [7] reported that a “passion for food” is a major driving force behind operating a food blog. Food bloggers have become opinion leaders in the food industry, and their recommendations significantly impact consumer behavior [8].

Cairns, Johnston, and Baumann defined foodies as individuals with a long-standing passion for eating and learning about food, but who are not food experts [9,10]. Foodies consider food not only a vehicle for biological nutrients but also a core part of their identity and lifestyle [9]. Food enthusiasts are a growing demographic worldwide and are a crucial target market for the food service industry. They are passionate about seeking and enjoying high-quality cuisine [9] and take pleasure in savoring and exploring food for extended periods [10]. Foodies generally enjoy sharing their dining experiences and cooking techniques on social media platforms. Accordingly, content that provides access to virtual gourmet culture is being created and maintained on social media, and it can be inferred that gourmets and social media are closely related. In particular, these social media are used as a very important source of information among young users, and the young generation make up the largest user base [11].

Generation MZ is a combination of Millennials (born in the 1980s) and Generation Z (born after 1995) and refers to today’s 20–30-year-olds [12]. They are digital natives who can freely use new digital technologies such as the Internet and mobile phones and have grown up on the Internet and social media [13]. They also have a high affinity for social media, a strong interest in food, and a passion for expressing views and sharing new foods on social media [6,14]. As a group of consumers, they have influenced market supply for several years [15]. As Generation MZ attract attention as powerful consumers because of their strong purchasing power [12], learning and understanding how to position food-related behaviors and the preferences of this generation is important for their segmentation into target markets. From a marketing perspective, the younger generation’s heavy reliance on social media plays an important role in generating sales and profits [16].

The characteristics of gourmets have been investigated in some previous studies. Robinson and Getz [17] studied the characteristics of Australian gourmet travelers, in which the participants were typically well-educated women with relatively high incomes who preferred to actively participate in experiences while traveling. In their in-depth investigation, Green et al. [5,18,19] found that self-identified foodie travelers tend to seek special types of food and follow local restaurants. A study conducted in the US [6] reported that the foodie tendency to share preferred dishes on social networks increases with age. Meanwhile, among studies related to foodie tendencies, although segmenting foodies into foodie destinations [19] has been investigated, these studies have mainly focused on gastronomy or gourmet characteristics in relation to tourism [9,14,17,18,19]. Pickering and Pickering [20] developed the 12-item Foodie Index, a measurement tool consisting of four subscales: enjoyment and interest in food, time investment, financial investment, and knowledge. This standardized tool is used to structurally evaluate foods. The overall scale and its subscales have shown excellent internal reliability and have clearly distinguished the differences in characteristics between gourmets and non-gourmets, helping to characterize these groups [20]. As the influence of social media has recently increased as an important marketing communication medium for sharing knowledge and experiences of food and dining out, research on gourmets’ use of social media has been conducted, mainly in food tourism-related topics. However, in terms of food and dining-out, few studies have examined the relationship between gourmet tendencies and social media use in depth. Some studies on social media related to dining out have been conducted; for example, authors have attempted to determine the motivation to eat out, the association of demographic characteristics [21,22], and the effects of social media characteristics on customer satisfaction and usage intention [23,24]. In addition, the impacts of image electronic word of mouth (eWOM) on fast food reviews [25] and Gen Y’s restaurant information-seeking and sharing behavior on social media [26] have also been studied. However, research on the relationship between the characteristics of food and dining out consumers and social media use is lacking. In particular, 97% of the population in Korea uses the Internet, and the country has the fastest Internet connection speed in the world [27]. Therefore, this study can be said to be even more necessary in the Korean context.

The main purposes of this study can be summarized as follows:

(1) To examine cooking and dining-out behaviors and social media use according to consumers’ foodie tendencies, focusing on Generation MZ in Korea
(2) To investigate the impact of foodie tendencies on social media use.

The results of this study are expected to provide valuable insights for understanding the consumer behavior characteristics of the younger generation in the food and dining-out industry and for establishing effective consumer segmentation and new marketing strategies for social media marketing.

## 2. Research Methodology

### 2.1. Data Collection and Sample

This study investigated the use of social media in relation to consumers’ foodie tendencies among consumers in their 20s and 30s with current social media accounts living in the Seoul–Gyeonggi region of South Korea. We selected these particular age groups because reports indicate that Korean consumers in their 20s and 30s show greater social media activity and engagement while exhibiting similar age-related social media behavior [28,29]. The participants were recruited via an online survey website (https://embrain.com) from 8–9 June 2023. We recruited 300 participants through an online panel in exchange for monetary compensation. The sample was representative of the Korean population in terms of gender and educational background. The study was conducted using convenience sampling. Only individuals who met the predetermined criteria were requested to participate in the survey. First, a guide explaining the study’s purpose and methodology was presented, and the survey was administered to those who agreed to participate. They were provided with structured online survey information, to which they responded. A total of 300 surveys were collected, of which 291 (97.0%) were included in the final analysis after eliminating nine insincere responses through data-cleaning procedures (147 female, 144 male). For ethical rigor, the research protocol describing the study’s purpose, content, and methods was approved by the Shinhan University Institutional Review Board (SHIRB-202305-HR-185-01).

### 2.2. Measures of the Study

A pilot survey was conducted one month prior to the final survey to identify and correct any ambiguous questions. To study the relationship between foodie tendencies and social media use, we extracted relevant questions from previous studies and reorganized them for this study. First, we used Pickering and Pickering’s (2022) Foodie Index [20] to assess participants’ foodie tendencies. A Korean version of the original English scale was developed through translation and back translation by three bilingual food experts. The Foodie Index consists of 12 items within four subscales: food enjoyment and interest, time investment, monetary investment, and knowledge. All scales were measured using a seven-point Likert scale (ranging from 1—strongly disagree to 7—strongly agree). To investigate the cooking and dining-out behaviors of consumers according to their food tendencies, the frequency of cooking/dining out/solo dining, the method of solo dining, and the average cost of dining out per person were investigated using a nominal scale. In addition, the frequency of these variables and the cost of dining-out were measured using seven-point (ranging from 1—rarely to 7—more than once daily) and six-point (ranging from 1—KRW < 1 million to 6—KRW > 5 million) Likert scales, respectively. To understand the attributes of social media use, questions about interest in social media; platforms used; average time spent on social media per day; frequency of checking social media; frequency of posting posts or photos on social media; restaurants searched for; and platforms used to search for food, cooking, and dining-out recommendations were assessed and scored on a nominal scale. In addition, three questions were asked about the extent to which consumers utilized social media recommendations when food shopping, cooking, and dining out, using a five-point Likert scale (ranging from 1—never to 5—always) [30]. Three questions regarding social media usage behaviors—active participation, provision of useful information, and regular posting—were evaluated using a seven-point Likert scale (ranging from 1—strongly disagree to 7—strongly agree) [31]. Additionally, information on demographic characteristics was collected, including sex, age, marital status, household composition, occupation, education level, and monthly household income, all of which were measured on a nominal scale.

### 2.3. Data Analysis

All statistical analyses were performed using IBM SPSS Statistics for Windows version 21.0 (Armonk, NY, USA). An exploratory factor analysis using principal components was conducted to categorize the survey participants according to their foodie tendencies using the Foodie Index and to evaluate its reliability and validity. The reliability of the extracted factors (via Cronbach’s α) was also analyzed. A K-means clustering analysis was performed on the factor scores derived from factorization to classify them into two groups. To characterize the two foodie groups, the mean scores for the two factors in each group were compared using the t-test. A frequency analysis was conducted to identify the demographic characteristics, cooking, and dining-out behaviors of the two groups of foodies, and chi-square tests were used to analyze the differences between the groups. In particular, questions regarding the frequency of cooking and dining-out, the average cost of dining-out per person, and frequency of eating alone were scaled to six or seven points, and differences in mean scores between the foodie groups were analyzed using the t-test. Likewise, frequency analysis and the chi-square test were used to analyze the differences between the foodie groups in terms of social media usage characteristics. In addition, the extent of social media usage, average daily social media usage time, social media checking frequency, social media posting frequency, and extent to which social media recommendations were used when purchasing food, cooking, and dining out were compared using the t-test by averaging the scores of the questions. Factor analysis was conducted on a questionnaire measuring the active use of social media, followed by a multiple regression analysis to examine the impact of foodie tendencies on the active use of social media.

## 3. Analysis and Results

### 3.1. Exploratory Factor Analysis and Clustering Based on Foodie Tendency

Table 1 presents the results of the exploratory factor analysis to evaluate the reliability and validity of the scales for the Foodie Index utilized in this study. The results of the principal component analysis, which were used to extract a common factor, showed that the factor loadings of all items were >0.6; these were extracted into two factors, and eigenvalues greater than 1 were extracted. The Cronbach’s α coefficients were 0.939 and 0.889, respectively, indicating a high degree of internal consistency. The calculated composite reliability values were 0.838 and 0.797, respectively, showing excellent values of over 0.7. The Kaiser–Meyer–Olkin (KMO) value was 0.905, indicating a good fit for factor analysis, while the Bartlett’s test χ^2^ was significant at 2715.292, with an explanatory power of 72.279% of the total variance. The two extracted factors were categorized as “foodie knowledge and spending” and “food interests and time commitments” based on the properties of each variable. A K-means cluster analysis was conducted using two-factor scores obtained by factorizing the Foodie Index to classify the participants into two distinct foodie groups. To determine the characteristics of the two foodie groups, the average scores for each factor were compared using *t*-tests (Table 2). The results showed that the high-foodie-tendency group scored significantly higher than the low-foodie-tendency group on both factors (*p* < 0.001). Several other studies have categorized food-related consumer groups; for example, Gunarathne et al. [8] identified six groups: passionate, interested, moderate, traditional, light, and non-foodies. Kline et al. [19] categorized foodie travelers according to their level of interest in food-related activities as enthusiasts with a very strong interest, creators with a neutral interest, and disinterested samplers. Additionally, Casini et al. [32] discussed the reduction of meal preparation time by categorizing individuals into “quickies” who prioritize time-efficient meal preparation, “foodies” who enjoy cooking, and “indifferent” consumers who are indifferent to meal preparation. As the present study specifically examined how foodie tendencies affect the use of social media rather than consumer group segmentation, two groups with different characterstics of foodie tendencies were compared.

### 3.2. Demographics According to Foodie Groups

The demographics did not differ significantly between the foodie groups (*p* > 0.05) (Table 3). The high-foodie-tendency group had a greater percentage of female participants, whereas the low-foodie-tendency group had a higher percentage of male participants. Additionally, participants in their 20s were more commonly in the high-foodie-tendency group, whereas those in their 30s were more commonly in the low-foodie-tendency group. Both groups had high proportions of single individuals. Regarding household composition, most participants in both groups were married with children. Office work was the most common field in both groups. Regarding education, college graduates were the most frequent in both groups, and individuals earning KRW > 6 million per month were the most common.

### 3.3. Cooking and Dining-Out Behaviors According to Foodie Groups

Examination of the cooking and dining-out behaviors of the two groups according to their foodie tendencies (Table 4) revealed a significant difference in the frequency of cooking and the method of eating when dining alone (*p* < 0.001). The group with a high foodie tendencies was found to cook more frequently than the group with a low foodie tendencies. Regarding eating methods when dining alone, the high-foodie-tendency group significantly tended to prepare and cook ingredients themselves, whereas the low-foodie-tendency group exhibited a greater preference for alternative methods of conveniently consuming food aside from cooking. Kline et al. [19] indicated that the group most interested in gastronomy reported significantly higher scores on the cooking dimensions of enjoying cooking, trying new recipes, and being creative, indicating a trend similar to that of the present study. In a study that explored the willingness to pay a premium to save time in meal preparation, foodies reported that the more time it took, the more willing they were to pay for the product [32]. According to Getz and Robins [33], who profiled food tourists in Australia, food markets—such as farmers’ markets—were the food-related events most visited by foodies, and this interest in food stemmed from a sense of authenticity [18]. These foodies’ “upscale” cooking activities included shopping at specialty cookware/food stores, attending cooking classes, and reading about nutrition [17]. Although the frequency of dining out and the average cost of dining out per person did not differ significantly between the groups (*p* > 0.05), the high-foodie-tendency group dined out more often. Additionally, the high-foodie-tendency group spent more on dining out. According to Getz and Robinson [33], foodies enjoy fine dining in expensive restaurants, with 16.3% reporting having eaten at a fine restaurant ≥3 times in the past 12 months. Furthermore, a study conducted by a food and beverage marketing company in the US revealed that 93% of foodies cook at home daily, and the majority of them dine out only 2–3 times per month, thereby implying little association between foodie inclination and the frequency of dining-out [6].

### 3.4. Social Media Usage Characteristics According to Foodie Group

Table 5 presents the social media usage characteristics of the two groups based on their foodie tendencies. First, the level of social media use in the high group was significantly higher than that in the low group (*p* < 0.05). As for the areas of interest on social media, while the high group showed a greater interest in food and dining out (41.6%) than the low group (23.7%), the difference was not statistically significant (*p* > 0.05). In terms of preferred platforms, both groups were more likely to use Instagram and YouTube, with the high group using Instagram and the low group using YouTube more, indicating a significant difference (*p* < 0.05). The two groups did not differ significantly in terms of average daily social media usage (*p* > 0.05); however, the high group had a higher proportion of users who spent >2 h per day on social media. Furthermore, upon scaling this question to a seven-point system and comparing the average scores between the two groups, the high group showed significantly higher scores than the low group (*p* < 0.05). The high group displayed significantly higher rates of frequency of social media checking and article or photo posting between the two groups, (*p* < 0.05). In addition, the high group demonstrated significantly greater utilization of social media recommendations for purchasing food, cooking, or dining out than the low group (*p* < 0.001). In other words, the group with high foodie tendencies exhibited greater social media usage; longer usage times; more frequent checking and posting; and greater reliance on recommended information for food purchasing, cooking, and dining out. These findings indicate a significant relationship between consumers’ foodie tendencies and social media use. This result was consistent with that of the study by Kline et al. [18], which suggested that the foodie group was the most active in social media activities, including posting food-related social media posts and following food organizations or businesses on social media platforms. Regarding the most commonly searched restaurants, those near the users’ current location ranked highest in both groups. Instagram and Naver Blog were the platforms most frequently utilized platforms by both groups for food and restaurant searches, while YouTube was particularly popular among the high group when compared with the low group. Regarding regular platform usage, Instagram and YouTube were popular in both groups, whereas Naver Blog was primarily used to search for food and restaurant recommendations. Additionally, YouTube had a strong following among consumers with foodie tendencies. YouTube is widely used by gourmet-oriented consumers. In other words, the platform’s primary usage appears to vary depending on its intended purpose and consumer inclinations.

### 3.5. Active Usage of Social Media According to Foodie Groups

To examine the differences in active social media use behavior among the foodie groups, we conducted a factor analysis of the active social media use questionnaire, as shown in Table 6. All items had factor loadings >0.6 and were extracted into a single factor with an eigenvalue of 2.300. The Cronbach’s α coefficient was 0.845 (composite reliability = 0.768), indicating good reliability. The KMO value was 0.710, and the Bartlett’s test χ^2^ was significant at 384.176, indicating an explanatory power of 76.655% of the total variance. The average value of the question on the active use of social media was significantly higher in the high group (*p* < 0.001) (Table 7).

### 3.6. Effect of Foodie Tendencies on Active Use of Social Media

The analysis of the effect of foodie tendencies on active use of social media by adjusting for age and gender (Table 8) showed that foodie tendencies explained 21.8% (*R*^2^ = 0.218, Adj *R*^2^ = 0.208) of the active use of social media (*F* = 19.990, *p* < 0.001). Foodie knowledge and spending (*t* = 3.986) and food interests and time commitments (*t* = 3.527), which are factors indicating foodie tendencies, had statistically significant positive (+) effects on active social media use. Foodie knowledge and spending (*β* = 0.275) had a stronger effect on active social media use than food interests and time commitments (*β* = 0.238).

## 4. Discussion and Implications

### 4.1. Conclusions

This study explored consumers’ cooking and dining-out behaviors and social media usage according to their foodie tendencies and examined how foodie tendencies affect active social media usage, focusing on Generation MZ in Korea. Firstly, the group with high foodie tendencies had higher average scores on food knowledge, spending factors, food interest, and time commitment factors than the group with low foodie tendencies. Second, the group with high foodie tendencies cooked more frequently than the group with low foodie tendencies. Even when eating alone, the proportion of people who prepared and cooked the ingredients themselves was much higher. On the other hand, the frequency of dining-out and the average cost of dining-out per person did not differ significantly between the two groups, but both were higher in the high foodie group. Third, the analysis of the characteristics of social media use by foodie tendencies showed significantly higher levels of social media use, average daily use time, checking frequency, and use of social media recommendations when purchasing food, cooking, and dining out in the high group than in the low group. Thus, a strong correlation between consumer foodie tendencies and social media usage characteristics can be inferred. Among the Korean consumers in this study, Naver Blog (41.6%), Instagram (27.9%), and YouTube (24.9%) were the most popular platforms used by the high group when searching for food recommendations. Fourth, the analysis of the effect of foodie tendencies on the active use of social media, foodie knowledge, and spending had a relatively strong influence on active use of social media.

### 4.2. Theoretical and Practical Implications

The theoretical implications of this study are as follows. First, the results of this study are unique in that they demonstrate the relationship between MZ consumers’ foodie tendencies and cooking and dining-out behaviors. While some studies have examined foodie tendencies, few have examined their relationship with direct cooking behavior. Therefore, this study is the first quantitative study to analyze the degree of foodie tendencies and overall eating-related behavior of the Korean Generation MZ using a validated tool. Therefore, this study will be valuable as an initial study on foodie tendencies and will expand upon the existing literature. Second, this study is one of few to directly examine the relationship between foodie tendencies and social media use. With the development of social media, searching for and utilizing information have become parts of the daily routine, and food-related information is expected to strengthen the cooking behaviors of foodies. Moreover, the findings on the relationship between foodie tendencies and social media use revealed that gourmet inclinations had a significant positive influence on the active use of social media. In other words, foodies play an important role in the production and dissemination of information in food-related fields by not only passively using social media but also by creating their own food, cooking, and dining-out-related gourmet content and actively sharing it on social media. Although these research results are widely known to social media users, they have not been studied in the literature. Finally, in the above context, this study expands the boundaries of research on foodie tendencies and suggests a new direction for future research. In other words, consumers’ foodie tendencies can influence not only the diversity of food choices but also the production, acquisition, selection, and dissemination of food-related information and thus have the potential to be widely applied not only in the field of food but also in the fields of media and marketing. As interdisciplinarity is being implemented, practical research may go on to study food alongside other disciplines. In particular, as the influence of social media expands across all fields, it is necessary to develop new approaches to consumer behavior research in the field of food, and this study can provide important inspiration for future works.

This study also has several practical implications. Firstly, governments, businesses, and researchers should pay more attention to the influence and potential of foodie trends. This is because foodie propensity provides a basis for food/restaurant marketing as a meaningful indicator of which consumer segments to target. Given the ripple effect of the active use of social media by consumers with high foodie tendencies, these consumers are likely to play an important role in food and restaurant marketing. In particular, as they prefer to cook their own food, providing information to stimulate this desire can effectively promote new or premium food ingredients.

Second, this study sheds light on the social media behavior of foodie groups and provides inspiration for marketing strategies to reach them. In other words, companies need to identify the platforms that gourmets mainly use when searching for dining out/cooking/food recommendations, as discussed in this study, and provide so-called “instagrammable” product-related tips that can trigger their social media activities. The content created through the active use of social media by foodies will have a ripple effect on the general public (non-foodies), stimulating appetite through social media posts and images [34]. Finally, foodie tendencies represent an important criterion that distinguishes the characteristics of an opinion leader on food-related social media. As they are consumer opinion leaders, their content will be utilized for the commercial marketing of food and restaurant products, establishing purchase criteria for consumers and acting as a catalyst that can trigger consumption. In fact, 81% of the online population receives purchasing advice from friends and followers on social media, and 74% of those who have received such advice report that it influences their decisions [26,35]. In particular, when looking for food/dining out information, personal experiences, opinions, reviews, meal photos, and suggestions related to dining-out experiences, many people now consult social media more often than friends and relatives [36]. It is crucial for companies to identify which group these food-related opinion leaders are, and foodie tendencies are an excellent indicator for this.

### 4.3. Limitations and Future Research

This study has several limitations. First, because the study participants were limited to Generation MZ in Korea, this study’s results cannot be generalized to the Korean population as a whole, or even to people on other countries. In addition, there are limitations to the in-depth discussion of the results of this study, owing to the lack of research on foodie tendencies in related fields and the characteristics of social media users related to food and dining out. Accordingly, future research could expand the age range and cultures of the participants, examine the relationship between foodie tendencies and social media use behaviors, and compare their results with those of this study. In addition, the strong relationship between foodie tendencies and social media usage suggests that social media behavior could be a crucial marker of foodie tendencies and may facilitate the creation of a new tool to assess foodie tendencies. Furthermore, as social media has become an important medium in the food sector, a scale can be developed to categorize and identify consumers’ food-related social media usage patterns. As social media increasingly impacts the food service industry, this study analyzed how consumers’ foodie tendencies influence their cooking and dining-out behaviors and social media use and how foodie tendencies influence their active use of social media, with a focus on the Generation MZ in Korea. The findings can have meaningful implications for the food and restaurant industry, helping to understand younger generations’ consumer behavior and develop new marketing techniques for consumer segmentation and social media marketing.

## Figures and Tables

**Table 1:** Factor analysis of the Foodie Index.

	Attribute	Factor Loading	Eigenvalue	Variance	Cronbach’s α Composite Reliability
Foodie knowledge and spending	A significant proportion of my income is spent on dining in fine/gourmet restaurants	0.854	6.680	38.758	0.939 **0.838**
	A significant proportion of my income is spent on fine/gourmet foods and/or ingredients	0.875			
	A significant proportion of my income is spent on cookbooks, food magazines, and/or cookware	0.894			
	I consider myself to have high knowledge of new food preparation methods	0.856			
	I consider myself to have a high knowledge of new food ingredients and flavors	0.819			
Food interests and time commitments	I like food	0.769	1.826	32.128	0.889 **0.797**
	I am interested in new food preparation methods	0.776			
	I often watch food shows on television (e.g., Celebrity Chef)	0.687			
	I am very interested in restaurant reviews	0.740			
	I am interested in new food ingredients and flavors	0.710			
	Typically, I spend a significant amount of time preparing food/meals	0.680			
	I often spend a significant amount of time choosing ingredients for meals	0.617			
KMO = 0.905		Bartlett’s test χ^2^ = 2715.292			
Total cumulative = 70.886%		(*df* = 66, *Sig* = 0.000)			

**Table 2:** Foodie tendencies according to foodie group.

	High Group (*n* = 173)	Low Group (*n* = 118)	*t*-Value
Foodie knowledge and spending	3.91 ± 1.45 ^(1)^	2.52 ± 1.27	8.69 ***^,(2)^
Food interests and time commitments	5.65 ± 0.60	3.85 ± 0.83	21.60 ***

^(1)^ Mean ± SD. ^(2)^ *** *p* < 0.001.

**Table 3:** Participant demographics.

		Total (n = 291)	High Group (n = 173)	Low Group (n = 118)	χ^2^ Value
Gender	Male	144 (49.5) ^(1)^	81 (46.8)	63 (53.4)	1.21
	Female	147 (50.5)	92 (53.2)	55 (46.6)	
Age (years)	20s	146 (50.2)	89 (51.4)	57 (48.3)	0.28
	30s	145 (49.8)	84 (48.6)	61 (51.7)	
Marital status	Single	227 (78.0)	135 (78.0)	92 (78.0)	0.00
	Married	64 (22.0)	38 (22.0)	26 (22.0)	
Household members	Single household	66 (22.7)	40 (23.1)	26 (22.0)	13.08
	Couple	33 (11.3)	22 (12.7)	11 (9.3)	
	Couple + children	150 (51.5)	81 (46.8)	69 (58.5)	
	Siblings	19 (6.5)	15 (8.7)	4 (3.4)	
	Other	23 (7.9)	15 (8.7)	8 (6.8)	
Occupation	Student	61 (20.7)	34 (19.7)	27 (22.9)	4.39
	Office worker	133 (45.7)	84 (48.6)	49 (41.5)	
	Professional	22 (7.6)	11 (6.4)	11 (8.8.3)	
	Service	27 (9.3)	14 (8.1)	13 (11.0)	
	None	19 (6.5)	14 (8.1)	5 (4.2)	
	Other	29(10)	16 (9.2)	13 (11.0)	
Education level	High school	30 (10.3)	11 (6.4)	19 (16.1)	7.67
	In college	43 (14.8)	25 (14.5)	18 (15.3)	
	College	197 (67.7)	123 (71.1)	74 (62.7)	
	Graduate school	21 (7.2)	14 (8.1)	7 (5.9)	
Average monthly income	KRW < 2 million	18 (6.2)	11 (6.4)	7 (5.9)	3.70
	KRW < 2 to 3 million	62 (21.3)	35 (20.2)	27 (22.9)	
	KRW < 3 to 4 million	46 (15.8)	24 (13.9)	22 (18.6)	
	KRW < 4 to 5 million	44 (15.1)	31 (17.9)	13 (11.0)	
	KRW < 5 to 6 million	35 (12.0)	22 (12.7)	13 (11.0)	
	KRW > 6 million	86 (29.6)	50 (28.9)	36 (30.5)	

^(1)^ N (%).

**Table 4:** Cooking and dining-out behavior by foodie group.

Attributes		High Group (n = 155)	Low Group (n = 136)	χ^2^ Value
Cooking frequency	Rarely	6 (3.5) ^(1)^	18 (15.3)	32.29 ***^,(2)^
	Once a month	5 (2.9)	13 (11.0)	
	Once every 2 weeks	9 (5.2)	11 (9.3)	
	Once a week	29 (16.8)	24 (20.3)	
	2–3 times per week	71 (41.0)	36 (30.5)	
	4–5 times per week	32 (18.5)	10 (8.5)	
	At least once a day	21 (12.1)	6 (5.1)	
Dining-out frequency	Rarely	5 (2.9)	7 (5.9)	6.93
	Once a month	13 (7.5)	15 (12.7)	
	Once every two weeks	30 (17.3)	23 (19.5)	
	Once a week	45 (26.0)	28 (23.7)	
	2–3 times per week	56 (32.4)	26 (22.0)	
	4–5 times per week	15 (8.7)	13 (11.0)	
	At least once a day	9 (5.2)	6 (5.1)	
Average cost of dining out per person	KRW < 100,000	18 (10.4)	20 (16.9)	4.63
	KRW < 100,000–200,000	46 (26.6)	33 (28.0)	
	KRW < 200,000–300,000	48 (27.7)	34 (28.8)	
	KRW < 300,000–400,000	34 (19.7)	175 (12.7)	
	KRW < 400,000–500,000	16 (9.2)	10 (8.5)	
	KRW ≥ 500,000	11 (6.4)	6 (5.1)	
Solo dining-out frequency	Rarely	16 (9.2)	14 (11.9)	4.62
	Once a month	10 (5.8)	9 (7.6)	
	Once every 2 weeks	6 (3.5)	7 (5.9)	
	Once a week	18 (10.4)	11 (9.3)	
	2–3 times per week	45 (26.0)	26 (22.0)	
	4–5 times per week	38 (22.0)	18 (15.3)	
	At least once a day	40 (23.1)	33 (28.0)	
How to dine alone	Trimming and cooking the ingredients yourself	67 (42.7)	18 (17.3)	22.59 ***
	Buying RTE ^(3)^ at the convenience store	9 (5.7)	7 (6.7)	
	Buying HMR ^(4)^, meal kit	39 (24.8)	31 (29.8)	
	Visiting restaurants	22 (14.0)	17 (16.3)	
	Delivery/Takeout	20 (23.7)	31 (29.8)	

^(1)^ N (%). ^(2)^ *** *p* < 0.001. ^(3)^ Ready-to-eat food. ^(4)^ Home meal replacement.

**Table 5:** Social media usage characteristics by foodie group.

		High Group (n = 173)	Low Group (n = 118)	*t*/χ^2^-Value
Social media usage scores ^(1)^		4.03 ± 1.12 ^(2)^	3.67 ± 1.28	2.54 *^(3)^
Areas of interest on social media	Food and dining out	72 (41.6) ^(4)^	28 (23.7)	13.69
	Fashion	25 (14.5)	19 (16.1)	
	Pets	10 (5.8)	10 (8.5)	
	Sports/exercise	17 (9.8)	15 (12.7)	
	Entertainment	27 (15.6)	31 (26.3)	
	Other	22 (12.7)	15 (12.7)	
Most used social media platform	Facebook	5 (2.9)	5 (4.2)	11.94 *
	Twitter	5 (2.9)	8 (6.8)	
	Instagram	105 (60.7)	66 (55.9)	
	Naver Blog	15 (8.7)	6 (5.1)	
	YouTube	36 (20.8)	31 (26.3)	
	Other	22 (12.7)	15 (12.7)	
Average daily social media usage time	<1 h	26 (15.0)	23 (19.5)	5.46
	<1–2 h	57 (32.9)	48 (40.7)	
	<2–5 h	70 (40.5)	40 (33.9)	
	<5–10 h	15 (8.7)	6 (5.1)	
	≥10 h	5 (2.9)	1 (0.8)	
Score for average daily usage time ^(5)^		2.51 ± 0.95	2.27 ± 0.86	2.26 *
Social media checking frequency	Less than once every few weeks	3 (1.7)	5 (4.2)	10.02 *
	A few times a week	12 (6.9)	9 (7.6)	
	1–2 times a day	23 (13.3)	27 (22.9)	
	3–4 times a day	20 (11.6)	19 (16.1)	
	Several times a day	115 (66.5)	58 (49.2)	
Frequency of posting articles or photos on social media	Not at all	23 (13.3)	27 (22.9)	8.38
	Once every few months	33 (19.1)	26 (22.0)	
	Once every few weeks	39 (22.5)	28 (23.7)	
	A few times a week	45 (26.0)	20 (16.9)	
	1–2 times a day	19 (11.0)	10 (8.5)	
	3–4 times a day	5 (2.9)	4 (3.4)	
	Several times a day	9 (5.2)	3 (2.5)	
Social media posting frequency score ^(6)^		3.32 ± 1.56	2.86 ± 1.54	2.46 *
Score for social media recommendation utilization for purchasing food ^(7)^		3.29 ± 1.05	2.63 ± 1.11	5.15 ***
Score for social media recommendation utilization for cooking		3.40 ± 1.12	2.78 ± 1.21	4.47 ***
Score for social media recommendation utilization for dining out		3.40 ± 1.13	2.86 ± 1.54	3.76 ***
Most searched restaurants	Restaurants near the current location	125 (72.3)	76 (64.4)	8.08
	Trendy restaurants	29 (16.8)	18 (15.3)	
	New restaurants	8 (4.6)	7 (5.9)	
	Others	9 (5.2)	9 (7.6)	
	Rarely searching	2 (1.2)	8 (6.8)	
Favorite SNS platforms for food and dining out recommendations	Instagram	48 (27.7)	34 (30.4)	2.47
	Naver Blog	72 (41.6)	53 (47.3)	
	YouTube	43 (24.9)	21 (18.8)	
	Other	10 (5.8)	4 (3.6)	

^(1)^, ^(5)^, ^(7)^ 5-point Likert scale. ^(2)^ Mean ± SD. ^(3)^ * *p* < 0.05, *** *p* < 0.001. ^(4)^ N (%). ^(6)^ 7-point Likert scale.

**Table 6:** Factor analysis for active use of social media.

	Attribute	Factor Loading	Eigenvalue	Variance	Cronbach’s α Composite Reliability
Active use of social media	Actively participating in social media	0.900	2.300	76.655	0.845 **0.768**
	Often providing useful content for other social network members.	0.833			
	Posting regularly on social media	0.892			
KMO = 0.710		Bartlett’s test χ^2^ = 384.176			
Total cumulative = 76.655%		(*df* = 3, *p*-Value = 0.000)			

**Table 7:** Active use of social media according to foodie group.

	High Group (n = 173)	Low Group (n = 118)	*t*-Value
Active social media usage	4.21 ± 1.52	3.39 ± 1.27	5.04 ***^(1)^

^(1)^ *** *p* < 0.001.

**Table 8:** Effect of foodie tendencies on active use of social media ^(1)^.

Dependent Variable	Independent Variable	*β*-Value	*t*-Value	*p*-Value
Active use of social media	Foodie knowledge and spending	0.275	3.986	0.000
	Food interests and time commitments	0.238	3.527	0.000
*R* = 0.467, *R*^2^ = 0.218, Adj *R*^2^ = 0.208, *F* = 19.990, *p*-value = 0.000				

^(1)^ Age- and gender-adjusted.

## Data Availability

The original contributions presented in the study are included in the article, further inquiries can be directed to the corresponding author.
